# Does Bariatric Surgery Improve Faecal Incontinence? A Systematic Review and Meta-analysis

**DOI:** 10.1007/s11695-021-05360-7

**Published:** 2021-04-14

**Authors:** Fardowsa Mohamed, Megna Jeram, Christin Coomarasamy, Melanie Lauti, Don Wilson, Andrew D. MacCormick

**Affiliations:** 1 Counties Manukau Health, 100 Hospital Road, Onehunga, Auckland, 2025 New Zealand; 2 University of Auckland, 85 Park Road, Grafton, Auckland, 1023 New Zealand; 3 University of Otago, 290 Great King Street, Dunedin Central, Dunedin, 9016 New Zealand

**Keywords:** Bariatric surgery, Faecal incontinence, Roux-en-Y gastric bypass, Gastrectomy, Biliopancreatic diversion, Duodenal switch, Pelvic floor disorder, Gastrointestinal health, Obesity, Urinary incontinence, Pelvic organ prolapse

## Abstract

**Introduction:**

Obesity increases the risk of pelvic floor disorders in individuals with obesity, including faecal incontinence. Faecal incontinence (FI) is a condition with important clinical and psychosocial consequences. Though it is associated with obesity, the effect of bariatric surgery on the prevalence and severity of FI is not well reported.

**Objective:**

To assess the effect of bariatric surgery on the prevalence and severity of FI in adult patients with obesity.

**Methods:**

This systematic review was conducted in accordance with the PRISMA statement. Two independent reviewers performed a literature search in MEDLINE, PubMed, Cochrane and Embase from 1 January 1980 to 12 January 2019. We included published English-language randomized control trials and observational studies assessing pre- and post-bariatric surgery prevalence or severity of FI. Random-effects models with DerSimonian and Laird’s variance estimator were used for meta-analysis.

**Results:**

Thirteen studies were included, eight assessing prevalence (678 patients) and 11 assessing severity of FI (992 patients). There was no significant difference in prevalence post-operatively overall, though it trended towards a reduction [pooled OR=0.55; *=*0.075]. There was a significant reduction of FI prevalence in women post-bariatric surgery [95% CI 0.22 to 0.94, *p*=0.034]. There was a statistically significant reduction in FI prevalence following Roux-en-Y gastric bypass and one anastomosis gastric bypass [0.46, 95% CI 0.26 to 0.81; *p*=0.007]. There was no significant reduction of incontinence episodes post-operatively [pooled mean difference =−0.17, 95% CI −0.90 to 0.56; *p*=0.65]. Quality of life (QOL) was not significantly improved post-bariatric surgery [mean differences for the following facets of QOL: behaviour −0.35, 95% CI −0.94 to 0.24; depression 0.04, 95% CI −0.12 to 0.2; lifestyle −0.33, 95% CI −0.98 to 0.33; *p* values of 0.25, 0.61 and 0.33, respectively].

**Discussion:**

There was a significant reduction in FI prevalence in women and those who underwent Roux-en-Y or one anastomosis gastric bypass. Our results for FI prevalence overall, FI severity and impact on quality of life were not statistically significant. Larger studies are needed in this under-researched area to determine the true effect of bariatric surgery on FI.

**Graphical abstract:**

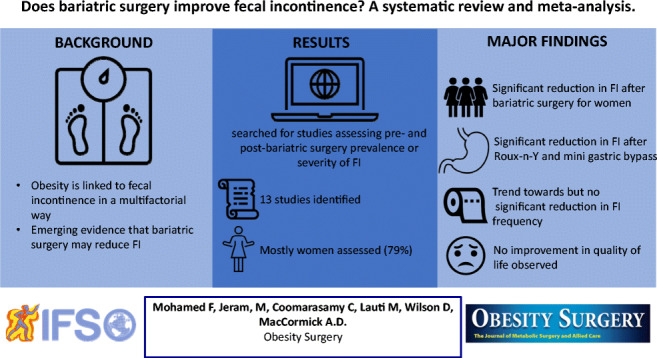

**Supplementary Information:**

The online version contains supplementary material available at 10.1007/s11695-021-05360-7.

## Introduction

FI is defined as the involuntary loss of liquid or solid faeces and, in some definitions, includes the loss of gas. Contributing factors include older age, female sex, an obstetric history, colorectal surgery and anorectal disease [1]. It is associated with other pelvic floor dysfunctions such as urinary incontinence (UI) and pelvic organ prolapse (POP) [2]. Obesity has recently been identified as an important contributor to FI [1]. Current literature suggests that FI prevalence may range from 16 to 63% in patients with obesity, whilst in the wider community, it may be 2.2 to 20.7% [3, 4].

The pathophysiological link between obesity and faecal incontinence is unclear and likely varies depending on the population assessed [4, 5]. An important pathophysiological cause of FI in obesity may be that obesity increases intra-abdominal pressure. One study of 110 obese women showed that the intraabdominal pressure was higher by 0.4 cm H_2_O per kg/m^2^ unit of BMI (95% confidence interval [CI] = 0.0, 0.7, *p* = 0.04)) [6]. Due to these high abdominal pressures, the anal sphincter recruits more motor units in obese peoples at baseline, therefore depleting muscle fibres available to recruit for additional demands, i.e. from loose bowel motions or pregnancy [7–10]. This increased anal sphincteric tone in obesity is demonstrated in multiple studies and may improve after weight loss and bariatric surgery, though results are variable in the literature [11–14]. In other populations such as in men and elderly patients, neurophysiological factors may play a role; however, the pathophysiology is poorly described in these groups.

FI can have a devastating impact on a person’s psychosocial quality of life, at times contributing to social isolation, embarrassment and depression, and thus ways of mitigating its effects are important to a patient’s well-being [2]. It also is shown to reduce independence in older people, and it is linked to increased admission to rest-homes. It also has a large financial impact on individuals and wider society. Drossman et al. surveyed 5400 US adults and found that 29.4% of those with large-volume FI, as opposed to 4.2% of non-FI adults, described themselves as too sick to work or go to school [12]. Considering the wide-reaching effects of FI on people and health systems, it is important to explore ways of reducing its prevalence and frequency in people with obesity.

Bariatric surgery may have an impact on FI, and it is important to elucidate this relationship as this may have a major impact on the patient’s post-operative well-being [10]. Poylin et al. conducted a systematic review assessing defaecatory disorders after Roux-en-Y gastric bypass surgery, including FI [4]. Across three studies, they found that the prevalence of FI reduced from 19.4 preoperatively to 8.6–9.1% post-operatively. Severity also improved, with symptom score reducing by 60%. Two other systematic reviews have examined pelvic floor dysfunction. Montenegro et al. conducted a systematic review assessing the impact of bariatric surgery on UI, POP and FI in obese women [11]. A non-significant 22% reduction in the rate of FI was identified. Lian et al. also assessed effect of bariatric surgery on UI, POP and FI, using validated questionnaires as their measure of outcome [15]. They found no improvement in FI after bariatric surgery across eleven papers. Both of the former reviews were confined to studies in female patients. Furthermore, both studies limited their search strategies to terms concerned with pelvic floor dysfunction, hence excluding studies that looked at gastrointestinal symptoms broadly. We conducted this systematic review to determine the effect of bariatric surgery on FI in both males and females with all available data.

## Methods

### Search Strategy

This systemic review was performed in accordance with the PRISMA statement [16]. Published, human-only articles from 1 January 1980 to 1 May 2019 were included. All randomized controlled studies, cohort studies and case series were included in analysis. Case reports, review articles, abstracts and systematic reviews or papers published in non-English languages were excluded from the final analysis.

Selection criteria for included studies were those that assessed the change in the prevalence and/or the severity of FI post-bariatric surgery in adult patients. The definition of FI as involuntary loss of stool and gas was used. Severity of FI was defined as the frequency of FI and/or its effect on quality of life. As there are multiple ways of measuring the prevalence and severity of FI, all measurement tools were accepted, including validated and non-validated questionnaires, and researcher-designed questionnaires. A follow-up time of greater than 6 months after surgery was included.

### Databases

The databases Ovid MEDLINE, PubMed, Embase and the Cochrane Library were systematically and simultaneously searched for articles regarding FI following bariatric surgery. Each databases’ MeSH terms were utilized, combining terms with the search functions “AND” and “OR”. Terms used in the search strategy are outlined in Table 1, and extensive search strategies for each database are displayed in Appendix 1.

**Table 1:** General characteristics of included studies

Title	Year of completion	Study design	Country	N	BMI difference	Age (mean)	T (months)	Surgical technique(s)	Primary outcomes	Other outcomes of the study
Ait Said	2016	Prospective cohort	France	116	11.9	43.6	12	RYGB and LSG	FI severity	UI
Burgio	2007	Prospective cohort	US	101	8.58	48.9	12	LRYGB	FI prevalence	
Cuicchi	2011	Prospective cohort	Italy	84		43.8	12	RYGB	FI prevalence	UI, POP
Elias	2017	Prospective cohort	Sweden	208	11.1	42.7	24	RYGB with and without BPD/DS	FI severity	Bowel habits
Sovik	2012	Randomised control trial	Norway	60	7	54.8	24	Lap bypass and duodenal switch	FI prevalence	GI symptoms, eating habits
Nickel	2016	Cross-sectional	Germany	186	13.7	46	24	SG, RYGB	FI severity	Gastrointestinal quality of life post-bariatric surgery
Fysekidis	2012	Cross-sectional	France	163	13.3	43.2	163	SG, LAGP	FI prevalence	nonspecific functional bowel disorders
Lee	2015	Prospective cohort	Taiwan	86	8.7	30.7	12	LSG, LRYGB and LMGBP	FI severity	
Whitcomb	2012	Prospective cohort	USA	98	11.8	39.71	12	Lap gastric banding, sleeve gastrectomy	FI prevalence, FI severity	UI, POP
Sileri	2011	Prospective cohort	Italy	68		47	6	SG	FI prevalence, FI severity	Constipation
Scorazzi	2013	Prospective cohort	Italy	32	9.5	46.3	15.6			UI, constipation
Romero-Talamas	2016	Prospective cohort	USA	72	10.5	47.5	12	SG, gastric banding, LGB	FI prevalence, FI severity	UI, POP
Bruzzi	2015	Prospective cohort	France	126	10	47	60	LMGB	FI severity	Gastrointestinal quality of life

### Study Screening and Selection

Two independent reviews (FM and MJ) conducted the search using the pre-designed search strategy (Table 1). Duplicates were manually deleted. As per the outlined inclusion and exclusion criteria, the two independent reviewers reviewed and extracted data from included papers. The reference lists of selected papers, and relevant reviews and systematic reviews, were also screened for relevant papers. If there were disagreements between the reviewers about included studies, a third reviewer (AM) was available to arbitrate. This was, however, not required.

### Outcomes

For the selected papers, the two independent reviewers extracted data for country of publication, study design, number of participants, surgical technique(s) used, mean age, sex, pre- and post-operative BMI and outcome tools used to measure FI frequency and/or severity. Outcome tools were used in the papers included in the systematic review: validated questionnaires, structured or semi-structured interview and independently created questionnaire’s where the questionnaire was available for viewing in the study. Separate meta-analyses and narrative reviews were done for the two outcomes. We conducted subgroup analyses by sex and type of bariatric surgery for FI frequency and severity. Where there was missing required data in included papers, the authors of the papers were contacted for further information.

### Quality Assessment

The Newcastle-Ottawa Quality Assessment Scale for cohort studies was used to assess the quality of studies by both reviewers [17]. The scale used applies a scoring system as follows: four points for participant selection, two points for comparability and three points for outcomes. The studies are classified as ‘poor’, ‘fair’ or ‘good quality’, depending on the total score out of nine, and the score in each of the three sub-categories. Studies of all qualities were included in the final analysis.

### Statistics

The meta-analysis was conducted for prevalence and severity of FI pre- and post-bariatric surgery. A random-effects model, with DerSimonian and Laird’s variance estimator and Mantel-Haenszel method, was used, and the results were presented as pooled odds ratio, with 95% confidence intervals (95% CI). A *p* value < 0.05 was considered statistically significant. Statistical heterogeneity among studies was assessed using Cochran’s Q test and the *I*^2^ statistic. All meta-analyses were performed using the R statistical software version 3.1.2.

## Results

**Fig. 1:**
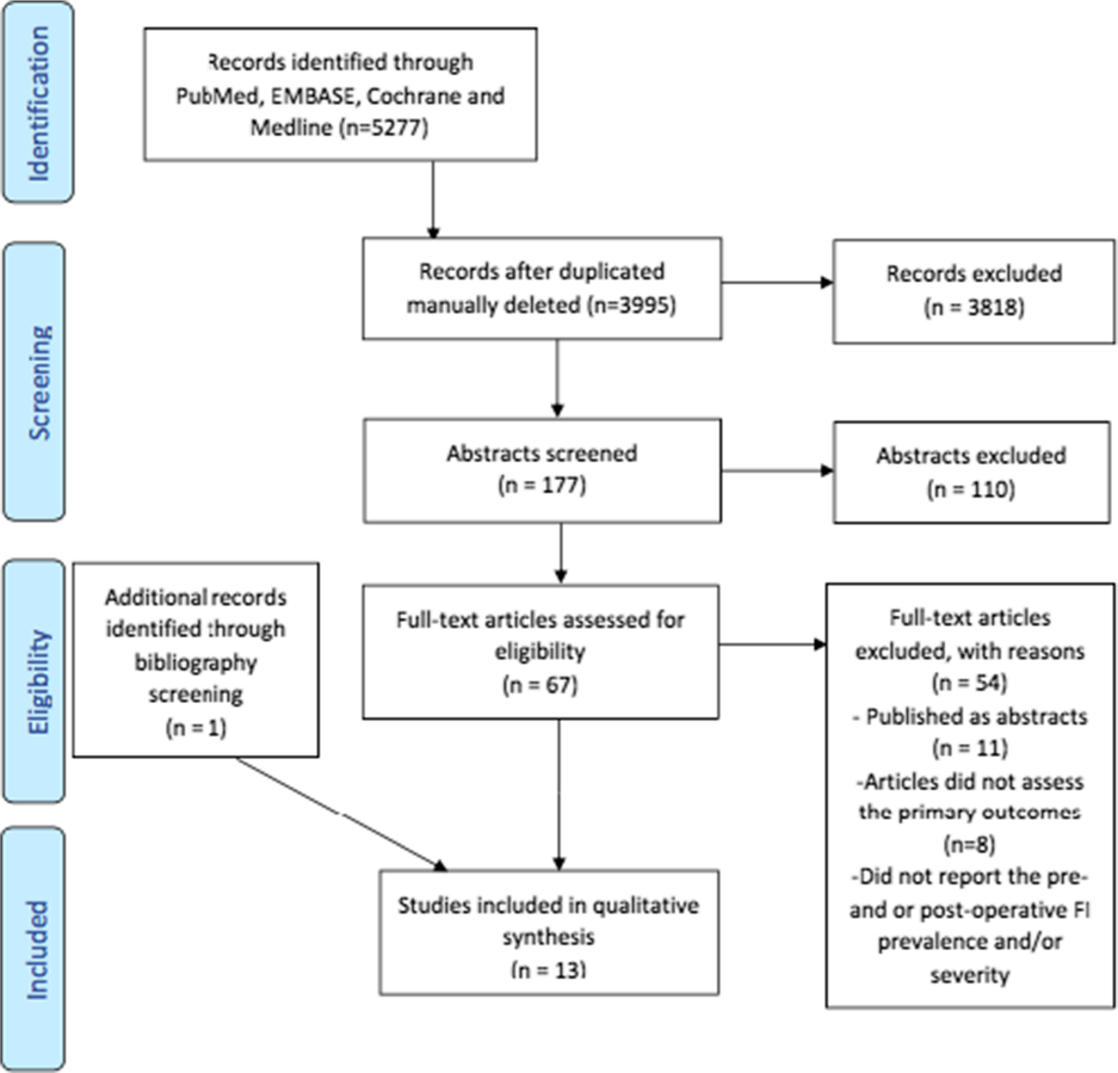
Study attrition diagram showing article count at each stage of the systematic review

After abstract screening, the review yielded 67 full-text papers, of which 12 met the inclusion criteria and a thirteenth paper was identified from manual review of bibliographies (Fig. 1).

The 13 included studies are summarized in Table 1. Eight articles assessed prevalence of FI pre- and post-bariatric surgery, and 10 assessed FI severity. A total of 1467 patients in total were included, of whom 1160 (79%) were women. Six hundred seventy-eight adults were assessed for prevalence and 1160 for severity. The mean age of patients was 42.8 years (range 30.7 to 54.8). The average follow-up time was 12.4 months for prevalence and 20.2 months for severity. Patients underwent five different types of bariatric surgery: 768 (53%) patients had Roux-en-Y gastric bypass, 190 (13%) had gastric banding, 177 (12%) had laparoscopic gastric sleeve, 166 (11%) had one anastomosis gastric bypass, and 29 (2%) had a duodenal switch. The surgery type was not specified in 137 (9%) patients.

### FI Prevalence Post-bariatric Surgery

**Fig. 2:**
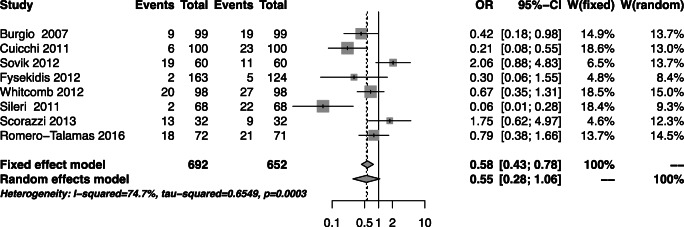
FI prevalence pre- and post-bariatric surgery

Of the 678 patients, 607 (89%) were woman. FI prevalence was measured in a number of ways across the eight papers; three studies used validated questionnaires, and five used researcher-designed questions. Of the validated questionnaire, two studies used the Faecal Incontinence Severity Index (FISI), and one used the Pelvic Floor Disability Index (PFDI-20). FI prevalence reduced from 24.5 to 20.9%, with an average follow-up time of 12.4 months [pooled OR =0.55, CI 0.28 to 1.06; *p*=0.075] (Fig. 2). The change in BMI was not significantly associated with prevalence of FI (*p*=0.24). We found that age was not significantly associated with the change in the prevalence of FI post-bariatric surgery (*p*=0.22).

### FI Severity Post-bariatric Surgery

**Fig. 3:**
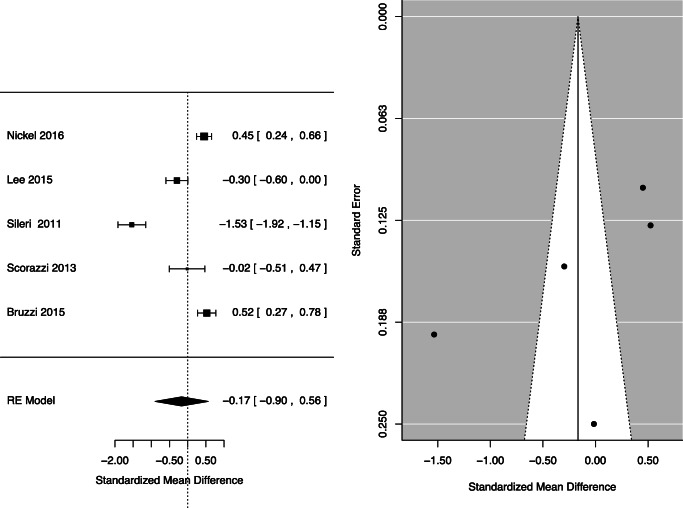
FI frequency pre- and post-bariatric surgery

Severity was assessed in 1393 patients, of whom 894 were woman (64%). All eight studies used a validated questionnaire to assess the severity of FI pre- and post-bariatric surgery. Three papers used the Faecal Incontinence Quality of Life Scale (FIQL) questionnaire, two used Gastrointestinal Quality of Life Index (GIQLI), two used the Wexner Scale, and one used PFDI-20. Five studies assessed pre- and post-operative frequency of FI episodes and found that frequency of incontinence episodes reduced post-operatively, though this was not a significant result [pooled mean difference =−0.17; 95% CI −0.90 to 0.56, *p* = 0.65] (Fig. 3).

**Fig. 4:**
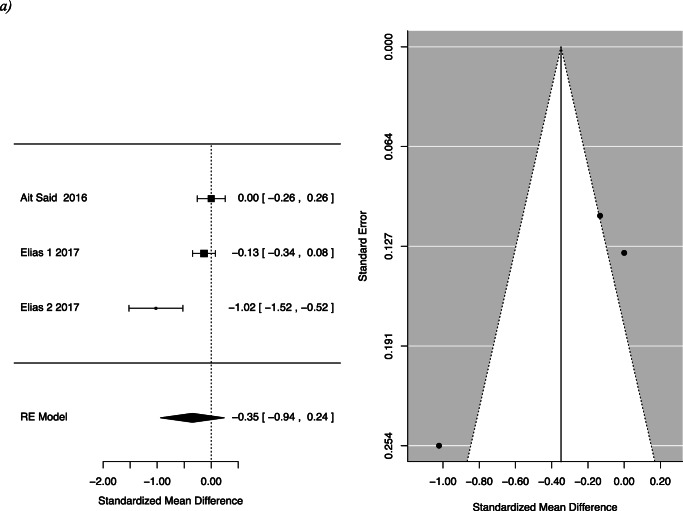

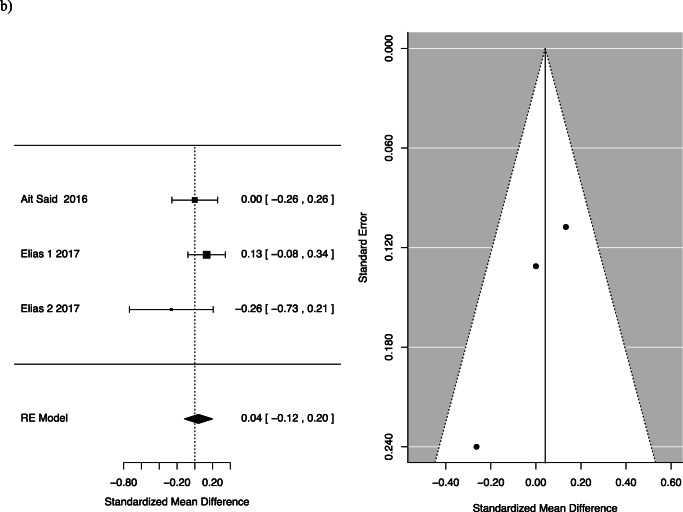

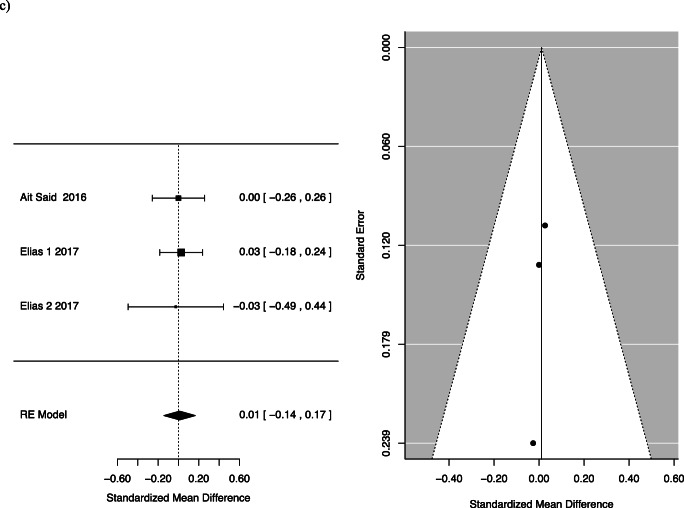

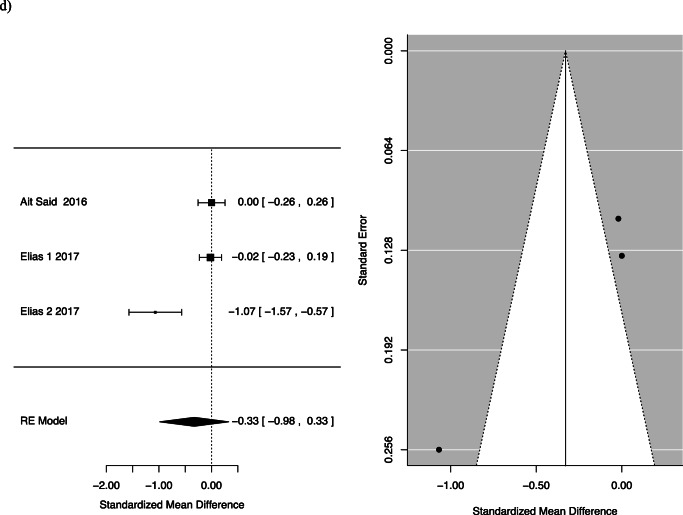
FI pre- and post-bariatric surgery quality of life: (**a**) behaviour, (**b**) depression, (**c**) embarrassment and (**d**) lifestyle

Two studies specifically assessed the quality-of-life impact using the FIQL questionnaire. One of the studies, Elias et al., separately analysed participants who received Roux-en-Y gastric bypass and duodenal switch, and so the two groups were considered separately [18]. Quality of life was divided into four facets by the questionnaire: effect on self-perception/depression, the effect on one’s lifestyle, associated embarrassment and coping. The mean differences for the following facets of QOL were as follows: behaviour −0.35, 95% CI −0.94 to 0.24; depression 0.04, 95% CI −0.12 to 0.2; and lifestyle −0.33, 95% CI −0.98 to 0.33 with *p*-values of 0.25, 0.61 and 0.33, respectively (Fig. 4).

### Change in FI Prevalence and Severity in Women

**Fig. 5:**
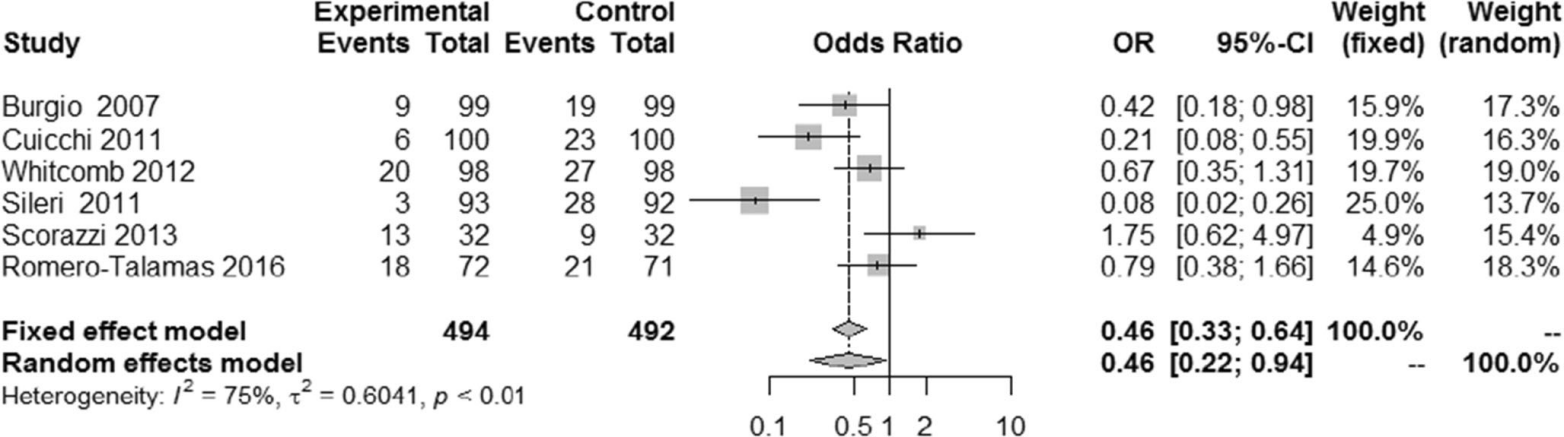
FI prevalence pre- and post-bariatric surgery in women

Of the 607 women in the meta-analysis for FI prevalence, sex-specific data was available for 492 women (81%). There was a significant reduction of FI prevalence in women post-bariatric surgery [0.46 (0.22 to 0.94); *p*=0.034] (Fig. 5). There was no statistically significant difference in FI severity frequency in woman pre- and post-bariatric surgery, though it trended towards improvement [0.66 [1.88 to 0.55], *p*=0.28].

### Change in FI Prevalence for Different Procedures

**Fig. 6:**
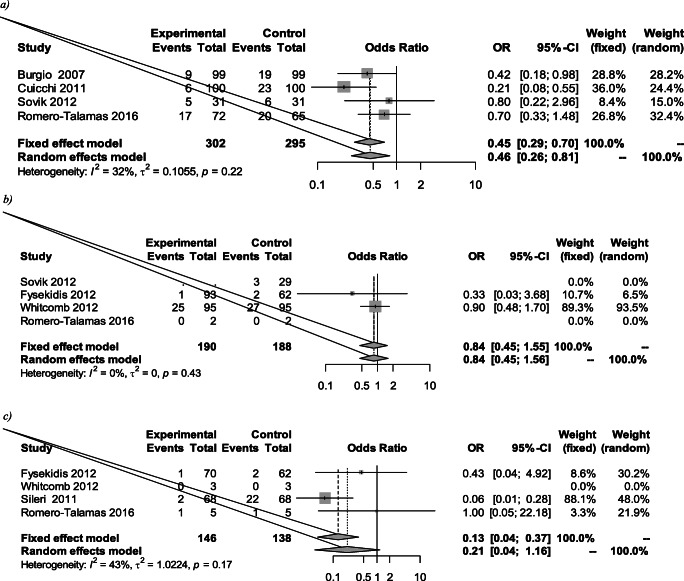
FI prevalence pre- and post-different bariatric procedures: (**a**) Roux-n-Y and one-anastomosis gastric bypass, (**b**) gastric banding and (**c**) sleeve gastrectomy

Of the 678, 390 (58%) underwent Roux-n-Y gastric bypass or mini-gastric bypass, 190 (28%) had gastric banding, 141 (21%) had laparoscopic or open sleeve gastrectomy, and 29 (4%) had a duodenal switch. There was a statistically significant reduction in FI prevalence following Roux-n-Y or one anastomotic gastric bypass [0.46 (0.26 to 0.81); *p*=0.007] (Fig. 6). There was no statistically significant reduction following sleeve gastrectomy or gastric banding [0.84 (0.45 to 1.56), *p*=0.59 and 0.21 (0.04 to 1.16), *p*=0.073, respectively].

### Quality Assessment

The studies were of varying quality and are displayed in Table 2. Of the 13 included papers, the average score out of nine on the Newcastle-Ottawa Quality Scale was 5.7 ± 1.4 (overall fair quality). Across the three domains, the averages were as follows: selectability 3.2 ± 0.6 out of 4, comparability 0.3 ± 0.5 out of 2 and outcome 2.2 ± 0.6 of 3. Four were marked as ‘good quality’, and the remaining nine were poor quality. All of the ‘poor quality’ papers were classed poor on the domain of comparability, due to a lack of a control group or analysis for confounding variables.

**Table 2:** Newcastle-Ottawa Quality Assessment Scale

				Newcastle-Ottawa Quality Assessment Scale			
Title	Year of study	Study design	Power calculation	Selection (out of 4)	Comparability (out of 2)	Outcome (out of 3)	Total assessment
Ait Said	2016	Prospective cohort	Absent	4	1	3	Good quality
Burgio	2007	Prospective cohort	Present	3	0	1	Poor quality
Cuicchi	2011	Prospective cohort	Absent	3	1	2	Good quality
Elias	2017	Prospective cohort	Absent	3	0	2	Poor quality
Sovik	2012	Randomised control trial	Present	3	0	2	Poor quality
Nickel	2016	Cross-sectional	Absent	3	0	2	Poor quality
Fysekidis	2012	Cross-sectional	Absent	3	0	2	Poor quality
Lee	2015	Prospective cohort	Absent	2	0	2	Poor quality
Whitcomb	2012	Prospective cohort	Absent	4	1	2	Good quality
Sileri	2011	Prospective cohort	Absent				
Scorazzi	2013	Prospective cohort	Absent	3	1	3	Good quality
Romero-Talamas	2016	Prospective cohort	Absent	3	0	2	Poor quality

## Discussion

This systematic review summarizes and reports available evidence on the effect of bariatric surgery on FI. For women, we found a statistically significant reduction in FI pre- and post-surgery [0.46; CI 0.22 to 0.94]. There was also a statistically significant reduction in patients after Roux-n-Y gastric bypass or one anastomosis gastric bypass. While there was a trend towards less FI following bariatric surgery among patients, this did not reach statistical significance [OR 0.55; CI 0.28 to 1.06; *p*=0.075]. The difference in the frequency of FI pre- and post-bariatric surgery was also not statistically significant. We assessed the effect of faecal incontinence on quality of life, across areas of behaviour change, depression, impact on lifestyle and feeling of embarrassment. We found no statistically significant difference pre- and post-operatively in these areas.

Faecal incontinence has important clinical and psychosocial consequences. It may contribute to loss of independence and significant psychosocial stress. Compounding this, it is currently underreported and likely underappreciated as a symptom [19]. For clinicians treating obesity with bariatric surgery, it is important to be able to communicate the effect bariatric surgery may have on their symptoms. This may be most important for women, for whom other risk factors for FI are often present such as obstetric history and perineal injury. This systematic review shows that women have a statistically significant reduction in FI prevalence post-bariatric surgery. Reduction of FI may therefore be an important consideration for obese women undergoing bariatric surgery

Our study found a statistically significant reduction of FI after Roux-en-Y gastric bypass and one anastomosis gastric bypass. This could be because Roux-en-Y represented the most common surgery in our studies, hence had the most power to reveal a significant result. Alternatively, Roux-en-Y gastric bypass may be particularly effective at reducing FI possibly through greater weight loss. We did not, however, find a statistically significant correlation between the reduction in BMI and reduction of FI, so there may be other hidden factors.

Though our conclusion regarding FI severity is limited by the heterogenous data, some insights can be gained from the included studies. The largest study that assessed quality of life after FI, Elias et al., looked at 208 men and women and found a statistically significant improvement in lifestyle, coping and behaviour and embarrassment after bariatric surgery [18]. Though we did not demonstrate a significant result, the results trended towards improvement in FI severity after bariatric surgery. Larger, standardized studies are needed to further elucidate this potential correlation.

We were not able to assess the pathophysiological factors that may be driving the reduction in FI after bariatric surgery. The literature suggests that obesity may increase intra-abdominal pressure and deplete the anal sphincter’s ability to remain continent. This evidence is particularly present in women, and thus reduced intra-abdominal and thus anal sphincteric pressure may explain the findings of our systematic review. However, our study showed that there is an improvement in FI after bariatric surgery independent of weight loss. We postulate that there are hidden factors driving the benefit of bariatric surgery on FI in women and gastric bypass patients.

We did not find an improvement in FI for men, or for patients undergoing other operations other than Roux-en-Y or one anastomosis gastric bypass. There were a small number of men and surgical procedures outside of gastric bypass in our study, so the study was perhaps not powered to detect these changes. In these patients, there may also be factors that are counteracting the benefits seen in women and bypass patients. Stool consistency is also an important determinant of continence. Elias et al. found a significant change in stool towards a looser consistency (*p*=0.04) in men and women after bariatric surgery. Diet is another important factor [18]. Dietary changes after bariatric surgery can work both to promote FI by loosening stool and improve FI through increasing stool bulk, so the relationship is difficult to investigate systematically. It could be that looser stool or diet changes affecting intestinal function counteract some of the benefits of bariatric surgery on FI, at least in some populations.

Systematic reviews hitherto have had varying results between bariatric surgery and improvement of FI. This is the first systematic review to assess both prevalence and frequency of FI in bariatric patients, in both men and women. A strength of our study is the assessment of severity of FI after bariatric surgery, which up to this point has not be assessed in a meta-analysis. Another strength of our systematic review included the broad search strategy, which identified papers that looked at pelvic floor disorders as well as those that looked at gastrointestinal disorders after bariatric surgery.

There are limitations to this systematic review and meta-analysis. Our study included patients with an age range of 30.7 to 54.8, and thus we cannot comment on faecal incontinence and the impact of bariatric surgery in older patients. As age is an important determinant in faecal incontinence, this is a key area for further exploration. The 13 studies were heterogeneous in the study design, population, surgery and outcome measurement tools. Importantly, there was a difference in how FI was defined; in one paper, Burgio et al. included gas loss as a form of incontinence [19]. On subgroup analysis in this paper, the FI was significantly improved if the definition was limited to liquid/stool loss. Regarding the assessment of severity, there was wide range of the measurement tools used, from validated questionnaires to researcher-made questionnaires. The measurement tools are thus of varying quality, even the validated ones. One commonly used validated questionnaire, GIQLI, is not specifically designed to detect FI and so has limited questions dedicated to FI symptoms [20]. Other questionnaires, such as FISI and the Wexner Scale, are designed for FI and so assessed the quality-of-life impact of FI with more rigor.

The papers included in meta-analysis were overall ‘poor’ quality in our assessment, and most papers lacked a control group or were not able to control for potential confounders. This impacts our ability to draw valid conclusions from these studies, as there may be confounding factors unaccounted for in our meta-analysis. There were limited studies evaluating FI in men, and so it remains unclear how FI and bariatric surgery impact male patients. Due to limited numbers and studies, we were unable to conduct a sub-group analysis for men. The papers were also not ethnically or geographically diverse, with most papers being based in Europe or in patients of largely European heritage. The studies had an age range of 30.7 to 54.8, and as FI is highly correlated with increasing age, our results would not be generalizable to older bariatric patients.

Further studies are required, particularly in older patients, male patients and multiple ethnic groups. As FI has large psychosocial, physical and financial impacts on patients, it may be an important pre- and post-operative symptom for some patients. By understanding how bariatric surgery will impact FI, surgeons and clinical teams can target treatments and manage expectations of their patients, thus providing better overall care.

## Supplementary Information

ESM 1 (DOCX 21 kb)
