# Supplementary material for: Does Bariatric Surgery Improve Faecal Incontinence? A Systematic Review and Meta-analysis
Source: Obes Surg. 2021 Apr 14;31(7):2942–53. doi: 10.1007/s11695-021-05360-7 (PMC8175321; doi:10.1007/s11695-021-05360-7)
Supplement: Supplementary file 1 — (DOCX 21 kb) [file 11695_2021_5360_MOESM1_ESM.docx]

*1 – Generic search terms used across databases*

| **Bariatric surgery** | **Faecal incontinence** |
| --- | --- |
| bariatric surgery | fecal incontinence |
| weight loss surgery | Feces incontinence |
| metabolic surgery | anal incontinence |
| gastric band | pelvic floor dysfunction |
| gastric bypass | pelvic floor disorder |
| vertical banded gastroplasty | incontinence |
| Roux-en-Y | CRADI-8 |
| duodenal switch | ICIQ |
| jejunoileal bypass | PFDI-20 |
| sleeve gastrectomy | FIQL |
| adjustable gastric band | FISI |
| biliopancreatic bypass | CRAIQ-7 |
| stomach bypass | GIQLI |
| Biliopancreatic diversion | PFIQ-7 |
|  | defecation habit |
|  | defecation disorder |
|  | defecation |
|  | bowel habit |
|  | constipation |
|  | flatulence |
|  | diarrhea |
|  | anorectal function |

*2 - Embase search strategy*

| Column 1 | Column 2 |
| --- | --- |
| 1# exp bariatric surgery/ | 24# exp feces incontinence/ |
| 2# bariatric surgery.mp. | 25# faecal incontinence.mp. |
| 3# weight-loss surgery.mp. | 26# anal incontinence.mp. |
| 4# biliopancreatic bypass.mp. | 27# incontinence.mp. |
| 5# exp biliopancreatic bypass | 28# exp pelvic floor disorder |
| 6# exp gastric band | 29# pelvic floor dysfunction.mp. |
| 7# exp gastric banding | 30# exp “ICIQ (incontinence)” |
| 8# gastric band*.m.p. | 31# CRAIQ-7.mp/ |
| 9# exp sleeve gastrectomy | 32# CRAIQ-8.mp/ |
| 10# sleeve gastrectomy.mp. | 33# PFIQ-7.mp/ |
| 11# exp gastric bypass surgery | 34# PFDI-20.mp/ |
| 12# gastric bypass.mp. | 35# FIQL.mp/ |
| 13# exp stomach bypass | 36# FISI.mp/ |
| 14# exp Roux en Y gastric bypass | 37# CRAIQ-7.mp/ |
| 15# Roux-en-Y.mp. | 38# GIQLI.mp/ |
| 16# duodenal switch.mp. | 39# PFIQ-7.mp/ |
| 17# exp jejunoilial bypass | 40# defecation habit.mp/ |
| 18# jejunoileal bypass.mp. | 41# exp defecation habit/ |
| 19# biliopancreatic diversion.mp. | 42# exp defecation disorder/ |
| 20# gastroplasty exp | 43# defecation disorder.mp/ |
| 21# gastroplasty | 44# exp constipation/ |
| 22# vertical banded gastroplasty | 45# constipation.mp/ |
| 23# stomach banding | 46# exp defecation |
|  | 47# defecation.mp/ |
|  | 48# exp diarrhea |
|  | 49# diarrhea.mp/ |
|  | 50# exp flatulence |
|  | 51# flatulence.mp/ |
|  | 52# Bowel habit.mp/ |
|  | 53#Anorectal function.mp/ |
| 54# 1 or 2 or 3 or 4 or 5 or 6 or 7or 8 or 9 or 10 or 11 or 12 or 13 or 14 or 15 or 16 or 17 or 18 or 19 or 20 or 21 or 22 or 23 | |
| 55# 24 or 25 or 26 or 27 or 28 or 29 or 30 or 31 or 32 or 33 or 34 or 35 or 36 or 37 or 38 or 39 or 40 or 41 or 42 or 43 or 44 or 45 or 46 or 47 or 48 or 49 or 50 or 51 or 51 or 53 | |
| 56# 54 and 55 | |

*3 – MEDLINE search strategy*

| Column 1 | Column 2 |
| --- | --- |
| 1 bariatric surgery.mp. | 21 exp Fecal Incontinence/ |
| 2 exp Bariatric Surgery/ | 22 faecal incontinence.mp. |
| 3 weight loss surgery.mp. | 24 anal incontinence.mp. |
| 4 metabolic surgery.mp. | 25 exp Pelvic Floor Disorders/ |
| 5 gastric band.mp. | 26 pelvic floor dysfunction.mp. |
| 6 gastric bypass.mp. | 27 pelvic floor disorder.mp. |
| 7 exp Gastric Bypass/ | 28 CRADI-8.mp. |
| 8 vertical banded gastroplasty.mp. | 29 ICIQ.mp. |
| 9 exp Gastroplasty/ | 30 PFDI-20.mp. |
| 10 Roux-en-Y.mp. | 31 FIQL.mp. |
| 11 duodenal switch.mp. | 32 FISI.mp. |
| 12 Jejunoileal bypass.mp. | 33 CRAIQ-7.mp |
| 13 exp Jejunoileal Bypass/ | 34 GIQLI.mp |
| 14 exp Gastrectomy/ | 35 PFIQ-7.mp |
| 15 sleeve gastrectomy.mp. | 36 defecation disorder.mp. |
| 16 adjustable gastric band.mp. | 37 bowel habit.mp. |
| 17 biliopancreatic bypass.mp. | 38 constipation.mp. |
| 18 exp Biliopancreatic Diversion/ | 39 exp Constipation/ |
| 19 biliopancreatic diversion.mp. | 40 flatulence.mp. |
| 20 stomach bypass.mp. | 41 exp Flatulence/ |
|  | 42 exp Diarrhea/ |
|  | 43 diarrhea.mp. |
|  | 44 anorectal function.mp. |
| 45 – 1 or 2 or 3 or 4 or 5 or 6 or 7 or 8 or 9 or 10 or 11 or 12 or 13 or 14 or 15 or 16 or 17 or 18 or 19 or 20 | |
| 46 – 21 or 22 or 23 or 24 or 25 or 26 or 27 or 28 or 29 or 30 or 31 or 32 or 33 or 34 or 35 or 36 or 37 or 38 or 39 or 40 or 41or 42 or 43 or 44 | |
| 44 – 42 and 46 | |

*4 – Pubmed search strategy*

("bariatric surgery"[MeSH Terms] OR ("bariatric"[All Fields] AND "surgery"[All Fields]) OR "bariatric surgery"[All Fields] OR (("weight"[All Fields] AND "loss"[All Fields] AND "surgery"[All Fields]) OR "weight loss surgery"[All Fields]) OR (("metabolic"[All Fields] AND "surgery"[All Fields]) OR "metabolic surgery"[All Fields]) OR (("stomach"[MeSH Terms] OR "stomach"[All Fields] OR "gastric"[All Fields]) AND banding[All Fields]) OR ("gastric bypass"[MeSH Terms] OR ("gastric"[All Fields] AND "bypass"[All Fields]) OR "gastric bypass"[All Fields]) OR (("stomach"[All Fields] AND "bypass"[All Fields]) OR "stomach bypass"[All Fields]) OR ("gastroplasty"[MeSH Terms] OR "gastroplasty"[All Fields] OR ("vertical"[All Fields] AND "banded"[All Fields] AND "gastroplasty"[All Fields]) OR "vertical banded gastroplasty"[All Fields]) OR "Roux en Y"[All Fields] OR (("duodenum"[MeSH Terms] OR "duodenum"[All Fields] OR "duodenal"[All Fields]) AND switch[All Fields]) OR ("jejunoileal bypass"[MeSH Terms] OR ("jejunoileal"[All Fields] AND "bypass"[All Fields]) OR "jejunoileal bypass"[All Fields]) OR (sleeve[All Fields] AND ("gastrectomy"[MeSH Terms] OR "gastrectomy"[All Fields])) OR "sleeve gastrectomy"[All fields] OR ("adjustable gastric band"[All fields] OR (adjustable[All Fields] AND ("stomach"[MeSH Terms] OR "stomach"[All Fields] OR "gastric"[All Fields]) AND "band"[All Fields])) OR ("biliopancreatic diversion"[MeSH Terms] OR ("biliopancreatic"[All Fields] AND "diversion"[All Fields]) OR "biliopancreatic diversion"[All Fields] OR ("biliopancreatic"[All Fields] AND "bypass"[All Fields]) OR "biliopancreatic bypass"[All Fields])) AND (((((((((("defaecation"[All Fields] OR "defecation"[MeSH Terms] OR "defecation"[All Fields]) AND ("habits"[MeSH Terms] OR "habits"[All Fields] OR "habit"[All Fields])) OR ("defaecation"[All Fields] OR "defecation"[MeSH Terms] OR "defecation"[All Fields])) OR (("defaecation"[All Fields] OR "defecation"[MeSH Terms] OR "defecation"[All Fields]) AND ("disease"[MeSH Terms] OR "disease"[All Fields] OR "disorder"[All Fields]))) OR (("bowel"[All Fields] AND "habit"[All Fields]) OR "bowel habit"[All Fields])) OR ("constipation"[MeSH Terms] OR "constipation"[All Fields])) OR ("flatulence"[MeSH Terms] OR "flatulence"[All Fields])) OR ("diarrhoea"[All Fields] OR "diarrhea"[MeSH Terms] OR "diarrhea"[All Fields])) OR ("anorectal"[All Fields] AND "function"[All Fields])) OR ((((((((((("faecal incontinence"[All Fields] OR "fecal incontinence"[MeSH Terms] OR ("fecal"[All Fields] AND "incontinence"[All Fields]) OR "fecal incontinence"[All Fields]) OR (("anal"[All Fields] AND "incontinence"[All Fields]) OR "anal incontinence"[All Fields])) OR (("pelvic floor"[MeSH Terms] OR ("pelvic"[All Fields] AND "floor"[All Fields]) OR "pelvic floor"[All Fields]) AND "dysfunction"[All Fields])) OR ("pelvic floor disorders"[MeSH Terms] OR ("pelvic"[All Fields] AND "floor"[All Fields] AND "disorders"[All Fields]) OR "pelvic floor disorders"[All Fields] OR ("pelvic"[All Fields] AND "floor"[All Fields] AND "disorder"[All Fields]) OR "pelvic floor disorder"[All Fields])) OR FISI[All Fields]) OR FIQL[All Fields]) OR PFDI-20[All Fields]) OR PFIQ-7[All Fields]) OR CRADI-8[All Fields]) OR CRAIQ-7[All Fields]) OR ICIQ[All Fields] OR GIQLI[All Fields] OR PFIQ-7[All Fields]))

*5 – Cochrane search strategy*

| **Column 1** | **Column 2** |
| --- | --- |
| #1 (bariatric surgery):ti,ab,kw | #20 (faecal incontinence):ti,ab,kw |
| #2 MeSH descriptor: [Bariatric Surgery] explode all trees | #21 MeSH descriptor: [Fecal Incontinence] explode all trees |
| #3 (weight loss surgery):ti,ab,kw | #22 (anal incontinence):ti,ab,kw |
| #4(metabolic surgery):ti,ab,kw | #23 (pelvic floor dysfunction):ti,ab,kw |
| #5 (gastric banding):ti,ab,kw | #24 (pelvic floor disorder):ti,ab,kw |
| #6 (stomach banding):ti,ab,kw | #25 MeSH descriptor: [Pelvic Floor Disorders] explode all trees |
| #7 (gastric bypass):ti,ab,kw | #26 (incontinence):ti,ab,kw |
| #8 MeSH descriptor: [Gastric Bypass] explode all trees | #27 (CRADI-8):ti,ab,kw |
| #9 (stomach bypass):ti,ab,kw | #28 (ICIQ):ti,ab,kw |
| #10 (vertical banded gastroplasty):ti,ab,kw | #29 (PFDI-20):ti,ab,kw |
| #11 MeSH descriptor: [Gastroplasty] explode all trees | #30 (FIQL):ti,ab,kw |
| #12 ("Roux-en-Y gastric bypass"):ti,ab,kw | #31 (FISI):ti,ab,kw |
| #13 (duodenal switch):ti,ab,kw | #32 (CRAIQ-7):ti,ab,kw |
| #14 (jejunoileal bypass):ti,ab,kw | #33 (GIQLI):ti,ab,kw |
| #15 MeSH descriptor: [Jejunoileal Bypass] explode all trees | #34 (PFIQ-7):ti,ab,kw |
| #16 (sleeve gastrectomy):ti,ab,kw | #35 (defecation habit):ti,ab,kw |
| #17 (biliopancreatic bypass):ti,ab,kw | #36 MeSH descriptor: [Defecation] explode all trees |
| #18 (biliopancreatic diversion):ti,ab,kw | #37 (defecation):ti,ab,kw |
| #19 MeSH descriptor: [Biliopancreatic Diversion] explode all trees | #38 (defecation disorder):ti,ab,kw |
|  | #39 (bowel habit):ti,ab,kw |
|  | #40 (constipation):ti,ab,kw |
|  | #41 MeSH descriptor: [Constipation] explode all trees |
|  | #42 (flatulence):ti,ab,kw |
|  | #43 MeSH descriptor: [Flatulence] explode all trees |
|  | #44 (diarrhea):ti,ab,kw |
|  | #45 MeSH descriptor: [Diarrhea] explode all trees |
|  | #46 (anorectal function):ti,ab,kw |
| #47: #1 or #2 or #3 or #4 or #5 or #6 or #7 or #8 or #9 or #10 or #11 or #12 or #13 or #14 or #15 or #16 or #17 or #18 or #19 | |
| #48: #21 or #21 or #22 or #23 or #24 or #25 or #26 or #27 or #28 or #29 or #30 or #31 or #32 or #33 or #34 or #35 or #36 or #37 or #38 or #39 or #40 or #41 or #42 or #43 or #44 or #45 or #45 or #46 | |
| #49 - #20 and #48 | |
